# Mapping the evolution of stigmatization in mental disorders: A bibliometric analysis from 1974 to 2024

**DOI:** 10.1007/s00127-025-03003-1

**Published:** 2026-02-24

**Authors:** Polat Goktas, Gul Dikec

**Affiliations:** 1 Faculty of Engineering and Natural Sciences, Sabancı University, Istanbul, Türkiye; 2 Faculty of Health Sciences, Department of Nursing, Fenerbahçe University, Istanbul, Türkiye

**Keywords:** Discrimination, Mental health, Bibliometric analysis, Social determinants, Social stigma

## Abstract

**Background:**

This bibliometric study scrutinizes the thematic evolution of research on stigma and discrimination in mental disorders, covering a span of five decades. It reflects on the shifting paradigms within the stigma-focused mental health research community from 1974 to 2024.

**Methods:**

A comprehensive bibliometric analysis was employed using the Bibliometrix R package and VOSviewer software, analyzing 1,892 articles from databases like Scopus, Web of Science, PubMed Central, and APA PsycInfo. Adherence to PRIBA guidelines ensured a holistic representation of the evolving research narrative.

**Results:**

The analysis outlined three distinct periods: the *Genesis Period* (1974 − 2007), focusing on foundational concepts of mental disorders and stigma; the *Growth Period* (2008 − 2015), which experienced a broadening into themes of discrimination and diagnostic refinement; and the *Rapid Growth Period* (2016 − 2024), characterized by a surge in research on child mental disorders and the impacts of posttraumatic stress disorder. Network analyses highlighted significant journals, key authors, and international collaborations that have shaped this field.

**Conclusions:**

The study maps a significant transformation in stigma-focused mental health research themes over fifty years, highlighting the growing complexity and the need for ongoing research into stigma and discrimination. It calls for interdisciplinary approaches to tackle these enduring challenges effectively.

**Supplementary Information:**

The online version contains supplementary material available at 10.1007/s00127-025-03003-1.

## Introduction

Erving Goffman, an American sociologist, defined stigmatization as a process that severely diminishes an individual’s self − worth by marking them with a trait or condition that drastically harms their reputation [21]. Goffman’s classic framework conceptualized stigma as a deeply discrediting attribute operating at the level of social interaction. Expanding on this, social psychologists Crocker and Quin highlighted stigmatization as the reduction in the value of social identity [14]. Link and Phelan [34] also introduced a broader perspective, asserting that stigmatization involves economic and political aspects and described the concept of power dynamics in stigma. According to their model, stigma progresses through a sequence of processes, from labeling and prejudice emergence to stereotyping, emotional responses, social exclusion, devaluation of social worth, and ultimately, discrimination [24]. In recent decades, the emergence of “structural stigma” theories has further deepened the discourse. Structural stigma refers to societal-level conditions, institutional policies, and cultural norms that systematically disadvantage individuals with mental disorders [25]. These contemporary models have catalyzed a shift in research attention, from individual-level prejudice to the broader social, economic, and political contexts that perpetuate stigma.

Notably, individuals with mental disorders have consistently been among the most stigmatized groups [27]. Public perceptions often associate mental illness with danger, unpredictability, or incompetence, labels that elicit fear, anger, or avoidance. These attitudes contribute to marginalization, social exclusion, and diminished access to quality care [12], [41]. Stigma continues to pose a significant challenge in mental health care, persisting even amid progress in the understanding and treatment of mental disorders, and is particularly pronounced in the context of conditions like schizophrenia [3]. To illustrate this theoretical progression, we have included Supplementary Table 1, which summarizes key definitional milestones from 1974 to 2024, aligning these shifts with thematic patterns identified in our bibliometric analysis. This table serves to contextualize the observed research trends within evolving scholarly understandings of stigma.

Stigmatization significantly undermines an individual’s confidence in their own abilities, especially for those diagnosed with mental disorders, where stigma acts as a major obstacle to seeking and adhering to treatment [1]. The fear of being stigmatized can cause people to interrupt or completely abandon their treatment plans [48], leading to frequent hospitalizations and a rise in health complications and mortality rates [36]. Consequently, there is a pressing need for research into the causes and consequences of stigma, personal experiences of stigmatized individuals, and measures to mitigate stigma, as well as assessments of the effectiveness of such interventions.

In response to the widespread and persistent consequences of stigma, global organizations have launched targeted campaigns to reduce its impact and promote stigma-focused mental health awareness. Responding to this need, the World Psychiatric Association initiated a global effort in 1998 to reduce mental disorder stigma, starting with the “*Opening the Doors*” campaign focused on schizophrenia, the condition most frequently associated with stigma. Following this initiative, several other campaigns like “*Changing Minds*” in England, “*Like Minds Like Mine*” in New Zealand, and the World Health Organization’s “*Comprehensive Mental Health Action Plan for 2013* − *2020*” launched in 2013 have been implemented to further combat stigma [9]. Additionally, research into stigma has broadened to include other groups facing discrimination in social sciences, criminology, and medicine, accelerating studies on the stigma related to HIV/AIDS [17], obesity [6], LGBTQ + individuals [47], and immigrants [10] over the past fifty years [9].

Recently, bibliometric analyses have become instrumental in examining stigma − related research trends, offering insights that aid mental health organizations and activists in strategic planning and advocacy [15, 16, 31, 47]. These analyses enable researchers to identify statistical frequencies, discern patterns, observe trends, and recognize biases, offering a comprehensive overview of the research field. Moreover, the insights gained from such analyses are valuable for international mental health organizations, institutions, and activists, aiding in the protection of human rights [47].

Thus, literature reviews reveal that bibliometric analyses in social and health sciences are increasingly focusing on trends within stigma − related research. These analyses specifically explore studies addressing stigma in certain groups [31, 32]. For instance, a bibliometric study on transgender health, covering research from 1990 to 2017, observed a significant rise in research activity, often linking the term “*transgender*” with HIV, mental health, and discrimination [47]. Similar analyses on HIV/AIDS have emphasized the role of stigma [17, 40]. Additionally, an evaluation of how mental disorders are depicted in media from 2002 to 2022 revealed a predominance of negative coverage, further intensifying the stigma around mental health issues [24].

However, existing bibliometric studies have not fully captured the evolving impact of global events like wars, traumas, and the pandemic on stigma research post − 2018. This oversight provides our study with an opportunity to use advanced bibliometric methods, through tools like the Bibliometrix R package and VOSviewer software, to thoroughly analyze articles from 1974 to 2024. The 50-year range was selected to capture the full historical arc of stigma research following foundational theoretical developments in the early 1970s, and to ensure bibliometric consistency across databases. This approach allows us to map out the thematic evolution of research on stigma-focused mental health, highlighting key thematic evolution phases,*Genesis*, *Growth*, and *Rapid Expansion*, and expanding the focus to a wider range of topics, including child mental health. We aim to address this research gap with a comprehensive, algorithmically enhanced analysis of the last five decades of stigma-focused mental health research. By outlining the thematic evolution and structural dynamics in this area, our study offers an in − depth look at how the focus of research has broadened from mental disorders and stigma to include discrimination, diagnostic refinement, and the impact of societal changes on stigma-focused mental health research. This detailed analysis not only fills a significant gap in the current literature but also lays a solid foundation for future research, policymaking, and the development of interventions to combat stigma and discrimination in mental health.

## Methods

Bibliometric methods analyze content and citations in scholarly articles to uncover trends, research dynamics, and intellectual structures across academic disciplines [19]. This approach, distinct from traditional literature reviews that gather, organize, and assess academic work within specific domains, offers a systematic and transparent framework [18]. Its primary advantage lies in revealing the relational, structural, and temporal dimensions of research streams, whether they are well − established or in emerging stages [29]. Such an analytical perspective enables a comprehensive understanding of the academic discourse’s evolution over time. Importantly, bibliometric studies does not necessitate ethical approval, as they involve analyzing publicly available scholarly publications without direct human participation. This aspect highlights the methodology’s efficiency and practicality in academic research synthesis. In executing this bibliometric investigation, the initial phase involved establishing a comprehensive review database. Subsequent phases entailed performing thematic evolution analysis and science mapping, aiming to elucidate the conceptual progression and identify prevailing thematic groupings within the research related to stigma and discrimination in mental disorders.

### Identification and selection of the documents

This study employs the PRIBA (Preffered Reporting of Bibliometric Analyses) framework, along with its scoping reviews extension by Koo & Lin [30], to systematically identify pertinent research on mental disorders. This standardized approach facilitates a thorough examination of relevant studies within the specified field.

A comprehensive bibliographic search was performed on scientific literature related to topic of discrimination and stigmatization in mental disorders. To achieve a broad and comprehensive literature coverage, we selected a diverse range of databases, Scopus, Web of Science, PubMed Central, and APA PsycInfo. Each database was chosen for its unique contribution to expanding the scope of literature across various relevant fields such as psychology, nursing, and social sciences, ensuring a thorough review of pertinent articles [4, 42]. To ensure data integrity and eliminate redundancy across these sources, we implemented a Python-based algorithm developed by the authors that automatically detected and removed duplicate records using matched metadata fields, including DOI, title, and author names. This deduplication process was essential to prevent skewed citation patterns and overrepresentation, and it resulted in a clean, unified dataset for subsequent bibliometric analysis.

To capture the extensive range of research in this area, we employed a systematic search strategy with carefully crafted search strings. These strings were designed to align with each database’s specific syntax and capabilities, utilizing keywords such as “*discrimination*,” “*stigmatization*,” and “*mental disorders*,” along with subject area filters and publication date limits. The full list of exact search strings used for each database is provided in Appendix A to ensure transparency and replicability.

The strategy to select the optimal search strings involved several steps:

- ***Broad Coverage***: Initially, broad keywords were used to ensure the inclusion of a wide range of relevant literature.
- ***Subject − Specific Focus***: Subsequently, searches were refined to target specific areas like psychology, nursing, and social sciences, using subject area filters where available to narrow down results.
- ***Keyword Variations and Synonyms***: To accommodate the diversity in terminology, synonyms and related terms were included in the search strings to capture studies that might use different language to describe similar concepts in the field of psychology.

Supplementary Table 2 outlines a structured approach to developing search strings for a comprehensive literature review. It begins with a broad sweep to ensure all relevant literature is considered, then narrows the focus to specific subject areas to refine the results, and finally broadens again to include synonyms and variations in terminology to capture the full spectrum of relevant research. These customized search strategies were aimed at conducting a comprehensive literature review, highlighting key themes, trends, and research gaps related to discrimination and stigmatization within mental disorders. The detailed search strings for each database and their outcomes are meticulously documented in Appendix A of the paper. The number of records obtained through each of these three approaches was compared and the third approach was found to deliver the most comprehensive set of records and was therefore used moving forward.

**Fig. 1:**
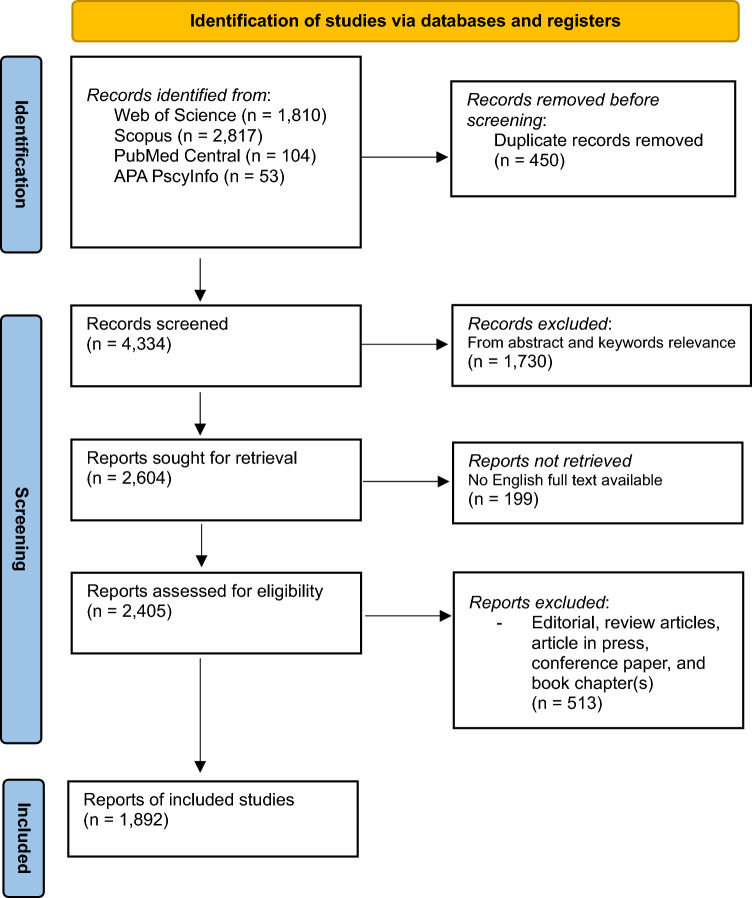
Flowchart of study selection process

The records were meticulously evaluated for their relevance and suitability for inclusion in the review, employing a structured critical appraisal process illustrated in Fig. 1. Initially, the title and abstract of each publication were examined to identify potentially relevant studies. Those that met the initial screening criteria were then subjected to a full − text review. To maintain methodological rigor in bibliometric analysis, inclusion and exclusion criteria were defined using metadata-driven parameters rather than solely content relevance. At the initial screening stage, records were selected based on bibliographic metadata, including titles, abstracts, keywords, and indexed subject areas, that aligned with predefined thematic domains: 1) analysis of discrimination and stigmatization in mental health, 2) detailed examination of these themes within stigma-focused mental health contexts, 3) exploration of interventions or programs addressing discrimination or stigmatization in mental health, or 4) critical discussions on the impact of such interventions in mental health settings. This approach ensured a comprehensive and focused review of the literature, aiming to capture a wide array of perspectives and insights on the subject matter.

Records were excluded from the analysis based on the following criteria, customized to the focus of our study on discrimination and stigmatization in mental health:

1. **Duplicate records** were removed to ensure uniqueness of data.
2. **Studies not examining discrimination or stigmatization** within the context of mental disorders were deemed irrelevant.
3. **Research outside the study’s thematic scope**, such as investigations not related to mental health (*e.g.*, studies focusing on physical health conditions without a mental health component), were excluded.
4. **Publications before 1974** were excluded to focus on more recent literature within the past 50 years, ensuring the contemporary relevance of the findings.

During a second stage of screening, the selected full − text articles were further evaluated, leading to the exclusion of items based on additional criteria:

1. **Lack of English full** − **text availability**, as the review was conducted in English.
2. **Studies with no in** − **depth examination of discrimination or stigmatization** related to stigma-focused mental health research, or only offering a superficial treatment of these issues.
3. **Misuse of terminology and concepts** indicative of low − quality research or problematic perspectives on stigma-focused mental health research, such as inappropriate references to “*panic*” or other stigmatizing language.
4. **Editorial, review articles, article in press, conference paper, and book chapter(s)** were excluded from direct analysis; however, their references were meticulously examined and considered for inclusion to ensure comprehensive coverage of the topic.

The screening and selection process was conducted independently by two reviewers (PhD) to enhance methodological rigor. Initially, titles and abstracts were screened to identify studies relevant to stigma and discrimination in mental disorders. For records that met the inclusion criteria or were ambiguous, full-texts were retrieved and assessed in detail. Disagreements between the two reviewers were resolved through discussion until consensus was reached. This collaborative approach ensured consistency and transparency in selecting the final dataset of 1,892 articles. This dataset encompasses citation and bibliographical details, abstracts and keywords, references, and information on funding for each of the publications, forming the basis for our comprehensive analysis.

### Bibliometric data analysis

This study embarked on a comprehensive bibliometric analysis to elucidate the developmental trajectory and thematic evolution of stigma-focused mental health research spanning five decades, from 1974 to 2024. Utilizing advanced bibliometric tools, we meticulously pointed through the extensive body of literature to unearth patterns, trends, and pivotal shifts in the domain of mental health research.

#### Data collection and preprocessing

We employed the Bibliometrix R package [2] and VOSviewer software [51, 52] for data visualization and analysis. The decision to use *Author Keywords*, rather than *Keywords Plus*, was intentional. Author Keywords reflect the authors’ conceptual framing and emphasize the article’s core focus, offering more targeted insight into the evolving discourse on stigma-focused mental health research [50]. In contrast, Keyword Plus terms, generated from titles of cited references, tend to capture broader and sometimes tangential concepts, which may dilute the thematic specificity required for our co-occurrence and trend analyses. Therefore, Author Keywords were prioritized to ensure conceptual alignment and analytical precision in this study. This approach facilitated a nuanced exploration of the stigma-focused mental health research domain, enabling us to identify and assess the performance of key scientific actors, discern conceptual and thematic evolutions, and pinpoint emerging research trends.

#### Data analysis plan

The data analysis in our study was structured in three distinct stages, each designed to incrementally build upon the findings of the previous stage to offer a comprehensive understanding of the stigma-focused mental health research domain. This structured approach leveraged the capabilities of the Bibliometrix R package and VOSviewer software, facilitating an in − depth examination of publication trends, conceptual evolution, and emerging research directions within stigma-focused mental health research.

#### Stage 1: Analysis of publication trends and performance of scientific actors

The first stage concentrated on analyzing the publication trends over the years and evaluating the performance of key scientific actors, including the most relevant authors and sources. By employing bibliometric indicators such as total citations and citation indices, we assessed the impact and contribution of these actors to the field. This step was crucial in understanding the intellectual structure of stigma-focused mental health research, identifying influential works, and highlighting the most active and cited scholars and journals within the domain.

#### Stage 2: Conceptual and intellectual network analysis

During the second stage, we utilized term co − occurrence and bibliometric coupling analysis through VOSViewer to explore the conceptual and intellectual landscape of stigma-focused mental health research. This involved mapping out the relationships between key terms and cited references to identify thematic clusters and intellectual networks. This stage illuminated the core themes that have shaped stigma-focused mental health research over the years, revealing how different concepts are interconnected and how these connections have evolved. It provided insight into the field’s theoretical foundations and identified the most prevalent and influential ideas driving scholarly discourse [8, 11].

#### Stage 3: Identification of emerging trends and future research directions

The final stage focused on the identification of emerging trends and the delineation of future research directions. Through a detailed review of the literature and application of thematic coding, we extracted and analyzed the main themes, research gaps, and methodological approaches within the collected body of work. This qualitative analysis facilitated the categorization of data into a cohesive thematic framework, highlighting areas ripe for future investigation [20]. By identifying unexplored and under − researched areas, this stage set the stage for future studies, proposing new research questions and suggesting directions that could contribute to the advancement of stigma-focused mental health research. Together, these stages formed a systematic and robust analytical framework that provided a multi − dimensional view of mental health research, enabling us to chart the field’s evolution, understand its current state, and envision its future trajectory.

## Results

### Descriptive statistics of bibliographic collection

A comprehensive analysis of the literature on this specific topic reveals that 1,892 documents have been published in 609 prominent journals. Throughout these publications, 31,091 keywords have been employed, with authors individually contributing 10,866 unique keywords. The articles were written by 7,574 unique authors, there being 4 co − authors on average per article with 12% of the articles being single − authored. There were 50,358 references in total, leading to an average citation rate of 26.63 per article. The collaboration index, which measures the average author engagement for each paper, is calculated at 4.79. Descriptive statistics summarizing the extant research on stigma-focused mental health research are detailed in Table 1. Furthermore, an examination of publication trends over the last 50 years indicates a steady increase in output, with an annual growth rate of 2.51% from 1974 (15 articles) to 2023 (124 articles).

**Table 1:** Descriptive statistics of bibliometric indicators for stigma-focused mental health research publications from 1974 to 2024

Metric	Description	Results
Documents	Total number of published documents on the topic	1,891
Sources	Number of unique journals and sources publishing these documents	609
Keywords Plus (ID)	Count of distinct ‘Keywords Plus’ used across the documents	31,091
Author’s Keywords (DE)	Count of unique ‘Author’s Keywords’ mentioned in the documents	10,866
Avg. Citations/Document	Average citations received per document	26.63
Authors	Total count of distinct authors contributing to these documents	7,345
Author Appearances	Number of times all authors appear across documents	8,138
Multi − authored Documents	Number of documents authored by more than one author	1,660
Single − authored Documents	Number of documents authored by a single author	229
Documents/Author	Average number of documents contributed by each author	0.257
Authors/Document	Average number of authors contributing to each document	4.327
Avg. Co − authors/Document	Average number of co − authors per document	4.332
Collaboration Index	Average number of collaborating authors per multi − authored document	4.792

### Bibliometric and network analysis

#### Key relevant sources

Table 2 showcases the leading journals in stigma-focused mental health research, ranked by their scholarly contributions and influence. This ranking is derived from a comprehensive analysis of bibliometric indicators, reflecting the journals’ academic impact and productivity in disseminating stigma-focused mental health research. This detailed examination of key journals based on robust bibliometric indicators like the number of publications, H − index, G − index, and total citation counts, offers a comprehensive view of their roles in advancing stigma-focused mental health research. Through this analysis, *‘Social Psychiatry and Psychiatric Epidemiology*’ emerges as a leading source, marked by its extensive contributions and widespread citation impact, closely followed by other pivotal journals such as ‘*Journal of Affective Disorders*’ and ‘*Psychological Medicine*,’ each contributing significantly to the field. When examining journals such as ‘*Stigma and Health*’ or ‘*Annales Medico-Psychologiques*’ with lower H − index and G − index values suggest they may be newer entrants in the field or their articles are yet to achieve wider citation recognition. Such insights are crucial for understanding the evolving landscape of stigma-focused mental health research publication venues.

**Table 2:** Summary of most relevant sources in stigma-focused mental health research

Source	No. of papers pub	H-index	G-index	Total no. of citations
Social Psychiatry and Psychiatric Epidemiology	104	42	68	5,039
Journal of Affective Disorders	91	30	48	2,674
Psychological Medicine	49	30	49	4,132
Journal of Clinical Psychology	23	10	20	407
Journal of the American Academy of Child and Adolescent Psychiatry	21	17	21	2,024
Journal of Child Psychology and Psychiatry	20	20	20	4,731
American Journal of Orthopsychiatry	20	14	20	762
Comprehensive Psychiatry	18	12	18	551
Annales Medico − Psychologiques	17	3	6	51
Stigma and Health	16	5	7	73

**Fig. 2:**
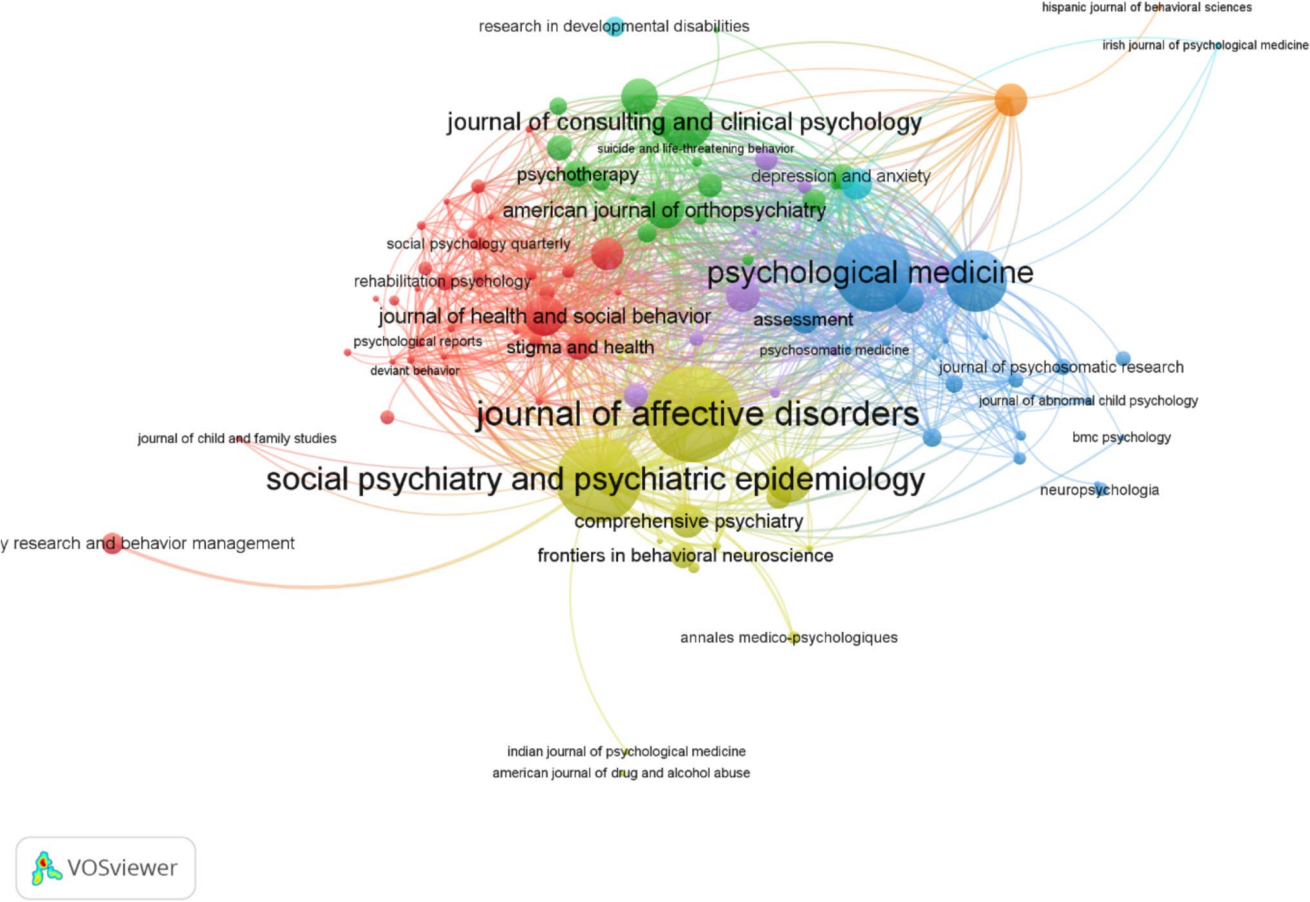
Network visualization of journal citations: This diagram depicts bibliometric coupling among journals using VOSviewer. Node size indicates the journal’s citation impact; line thickness shows coupling strength based on shared references. Colour − coded clusters highlight related journals within specific research domains

Furthermore, as Fig. 2 showcases, the landscape of stigma-focused mental health research is intricately mapped out through a bibliometric coupling network, where the significant repositories of academic discourse, the journals, are delineated into three distinct clusters. Each cluster is categorized in its own unique colour—yellow, red, green, or blue—signalling not just aesthetic differentiation but also thematic congruence within each group. Journals within the same cluster exhibit a harmonious relationship, often citing each other, reflecting a unified front in research themes and discourse. The fabric of this scholarly network is further defined by the links that bind these clusters; the spatial nearness of the nodes (each representing a journal) is telling of the bibliometric bonds they share—the closer the nodes, the stronger their connection. The volume of each node is proportional to the journal’s academic influence, as measured by citation counts. The ‘*Journal of Affective Disorders*’ stands out within this network, asserting its centrality in affective disorder research, while *‘Social Psychiatry and Psychiatric Epidemiology,*’ ‘*Psychological Medicine,*’ and ‘*Journal of Consulting and Clinical Psychology*’ are also prominent, contributing significantly to the collective scholarship in the mental health sciences domain.

#### Author influence

Table 3 presents the most influential scholars in the domain of mental health research, ranking them based on their scholarly outputs, citation impacts, and the breadth of their contributions over time. Leading this esteemed group, Corrigan P.W. stands out with an impressive portfolio of 18 published articles and a towering H − index of 134, alongside 1,345 total citations, marking a significant impact in the field since 1998. Following closely, Thornicroft G. and Evans-Lacko S. have both demonstrated considerable focus on stigma-focused mental health research, with each contributing 10 articles and securing total citations of 572 and 493, respectively, since 2012.

**Table 3:** Top ten most productive authors in stigma-focused mental health research

Author	H-index	G-index	TC	NP	PY_start_
Corrigan P.W	134	12	1,345	10	1998
Thornicroft G	57	12	572	10	2012
Evans-Lacko S	49	12	493	10	2012
Link B.G	109	10	1,093	8	1997
Schomerus G	25	10	257	8	2006
Rüsch N	20	10	206	8	2010
Jorm A.F	18	10	182	8	2009
Phelan J.C	112	9	1,121	7	1997
Angermeyer M.C	77	9	777	7	1997
Reavley N.J	4	9	47	7	2017

**Notes**: H-index (H): A metric that measures both the productivity and citation impact of the publications of an author. An author with an index of ‘h’ has published ‘h’ papers, each of which has been cited in other papers at least ‘h’ times. G-index (G): An index intended to improve upon the H-index by giving more weight to highly-cited articles. It represents the largest number ‘g’ such that the top ‘g’ articles received together at least ‘g^2^’ citations. Total Citations (TC): The aggregate number of citations that the author’s articles have received, reflecting the overall reach and influence of their research output. Number of Papers (NP): The total number of published articles attributed to the author within the field of stigma-focused mental health research. Publication Start Year (PY_start_): The year the author published their first article in the domain of stigma-focused mental health research, indicating the length of their academic contribution to the field.

This compilation underlines the diverse yet profound contributions made by each author, highlighting that both the volume of research and the depth of impact (as measured by H − index and G − index) play critical roles in advancing stigma-focused mental health research. It is evident from this analysis that impactful research can span across various topics within the mental health domain, with some authors focusing on specific areas or technologies and others contributing broadly across the field. The varied publication years further showcase the evolving nature of mental health research and the continuous contributions by both seasoned and newer researchers to this ever − important field.

#### Affiliation statistics

Supplementary Fig. 1 shows the results and displays only countries with significant influence, defined as those with a total link strength (TLS) above the median threshold (TLS > 1,500) based on co-authorship data. This filter was applied using VOSviewer to enhance readability and to focus on countries that play a central role in global stigma-focused mental health research collaborations. The size of each node within the network is directly proportional to the TLS of the respective country, which is a composite measure reflecting both the volume of research output and the intensity of international collaborations. The United States emerges as the pivotal node, signifying its central role in the global research landscape, with a pronounced density of linkages to numerous countries; 20,742 TLSs. This prominence is indicative of the country’s extensive involvement in international research and its substantial contribution to the global knowledge pool. In the vicinity of this central hub, other nations also manifest their influence through substantial node sizes and TLSs: the United Kingdom with 12,748, Germany with 7,927, Canada with 5,757, and China with 2,857. These figures not only highlight the countries’ active roles but also their strategic positions as key intermediaries in the network, suggesting their capacity to facilitate a substantial flow of knowledge across borders. These countries, alongside the United States, emerge as primary conduits for scholarly exchange, bolstering the notion that research is a collaborative enterprise that transcends geographical and disciplinary boundaries.

The six color − coded clusters delineate the nodes into distinct groups, potentially reflecting geographical proximity or thematic alignment in research efforts. For instance, European countries tend to aggregate, suggesting a regional predilection for collaboration, possibly fostered by shared funding programs and research initiatives. Inter − node connections, visualized by the lines between countries, vary in thickness, which represents the relative volume of interconnections. Thicker lines denote a stronger bibliometric link, typically a result of higher collaborative activities, such as co − authored publications. Thus, this network visualization not only highlights the central players within the international academic community but also provides insights into the structure and dynamics of research collaboration on a global scale in the stigma-focused mental health field.

#### Intellectual structure of stigma-focused mental health research narrative

**Fig. 3:**
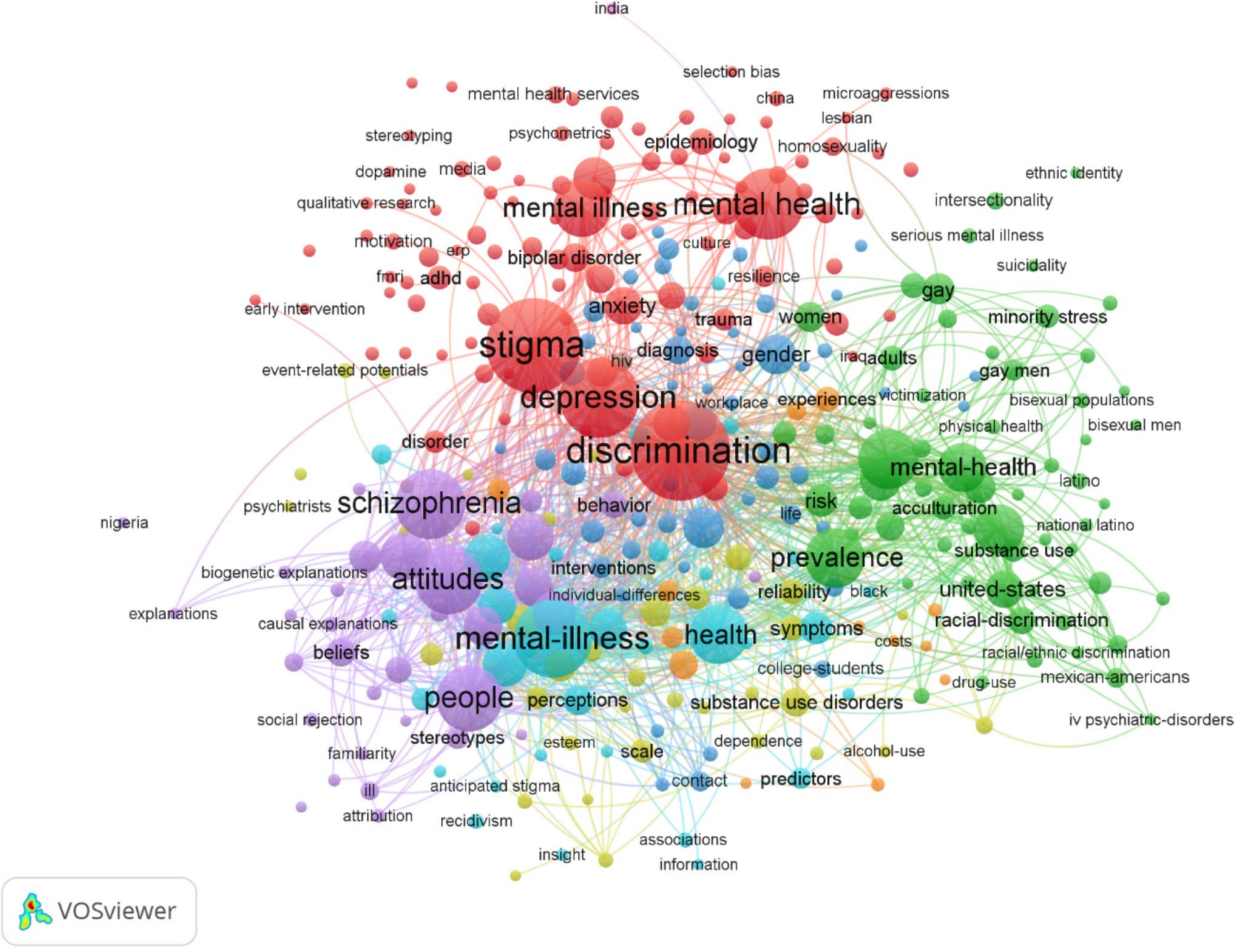
Co − occurrence analysis of most frequently − occurring author keywords

In order to further explore prevailing trends and emerging areas within the field, we performed a co − occurrence analysis of author keywords utilizing VOSViewer, as depicted in Fig. 3. By examining the frequency and patterns of keyword appearances across publications, we can discern the thematic contours that are currently shaping scholarly inquiry.

In the context of the provided figure, which presumably synthesizes a large number of articles within the field of stigma-focused mental health research, the keywords serve as indicators of thematic concentration. For instance, prominent terms such as *discrimination* (Occurrences: 267), *stigma*, (245), *depression* (175), *mental* − *illness* (173), *mental health* (144), *schizophrenia* (137), *attitudes* (128), *people* (124), *prevalence* (104), *psychiatric* − *disorder* (104), *health* (101) and *mental illness* (95) highlight key areas of focus within the field. The number of occurrences of each keyword, while not explicitly provided, would follow a similar pattern to the one seen in the nursing research field example, offering a quantifiable measure of each theme’s prominence.

The network mapping of these keywords, as illustrated in the Fig. 3, enables the visualization of the co − occurrence strength—that is, the frequency with which pairs of keywords appear together within the same articles. Thicker lines connecting the keywords indicate a higher number of co − occurrences, signifying stronger associative links between the concepts they represent. Clustering algorithms within the VOSViewer software further enhance our understanding by segregating keywords into colour − coded clusters. These clusters represent thematic subfields or closely related concepts within the broader research landscape, where a higher density of intra − cluster connections suggests a more cohesive thematic area. For example, a cluster might group together terms related to specific stigma-focused mental health conditions and their societal perceptions, such as “*stigma*,” “*schizophrenia*,” “*discrimination*,” and “*attitudes*.” Here, keywords that frequently appear together in the literature are clustered by colour, indicating thematic concentrations within the dataset.

In this visualization, the primary cluster (red) aggregates keywords that pertain to mental disorders and the social perception of stigma-focused mental health, including *stigma*, *depression, discrimination, mental illness, mental health, anxiety,* and *mental disorders*. This cluster emphasizes the social and emotional facets of stigma-focused mental health, pointing to a significant research focus on the societal impact and understanding of these conditions. The second cluster (green) compiles keywords related to the demographic and social determinants of mental health, such as *prevalence, psychiatric* − *disorders, perceived discrimination, risk, racial* − *discrimination, gay, ethnic* − *identity, black, latino* and *united* − *states*. These terms indicate a focused exploration into the variations of mental health issues across diverse populations, particularly concerning sexual orientation and ethnic identity, as well as their intersections with sociocultural contexts. A third cluster (blue toned versions) encompasses terms related to cognitive aspects and severe mental health conditions, including *schizophrenia, attitudes, prejudice, mental* − *illness, mental* − *disorders, bias, beliefs* and *people*. This grouping reflects the research interest in the cognitive and societal understanding of mental health, particularly severe conditions and their implications for identity and perception. Lastly, the yellow cluster focuses on specific aspects and populations within mental health research: *drug* − *addiction, drug* − *users, substance use disorders and alcohol*. These terms highlight a focus on how mental health intersects with substance use disorders.

**Fig. 4:**
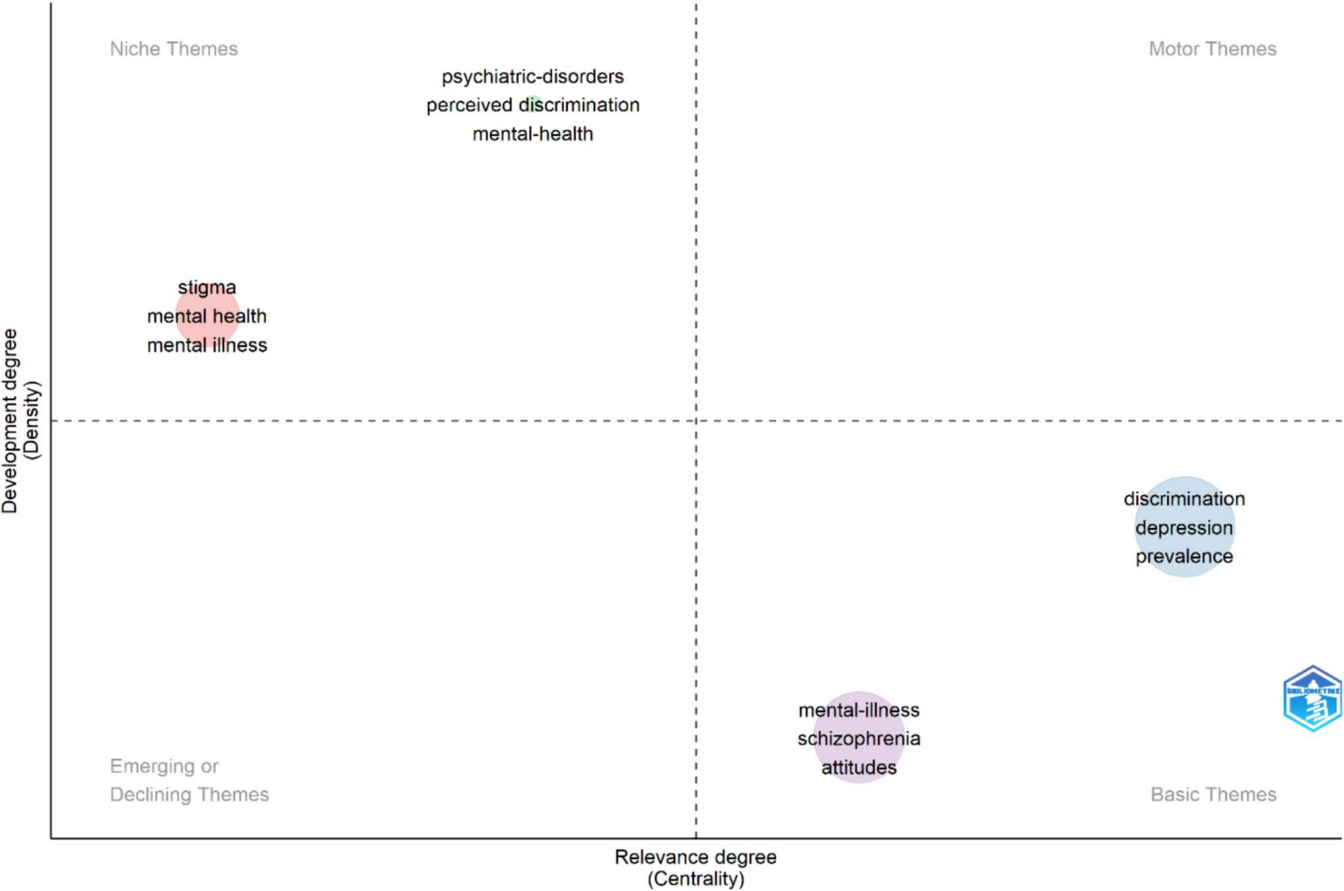
Thematic map of author keyword co − occurrence in stigma-focused mental health research narrative

Furthermore, as presented in Fig. 4, the bidimensional mapping offers a nuanced classification of keyword clusters, organized by their degree of centrality (relevance) and density (development), which delineates the thematic structure within stigma-focused mental health research [11]. In the current mapping, ‘stigma,’ ‘mental health,’ and ‘mental illness’ are positioned within the Niche Themes quadrant. This indicates that these topics are well-developed and have a cohesive body of literature but occupy a more specialized role in the overall research landscape, with comparatively lower centrality than Basic or Motor Themes. Keywords with low centrality and low density are characterized as “*Emerging or Declining Themes*.” These represent areas that are either nascent in their academic journey or diminishing in scholarly focus. On the contrary, keywords that possess high centrality but low density are recognized as “*Basic Themes*.” These are well − established, foundational topics within the field that continue to underpin ongoing research. The “*Niche Themes*” are identified by their low centrality but high density, indicating specialized areas of research that, while perhaps limited in their centrality to the broader field, have a well − developed body of work and a tight − knit scholarly community. Conversely, “*Motor Themes*,” marked by both high centrality and high density, are the driving forces of the research area, indicating themes that are not only mature but also central to the field’s scholarly discourse.

The placement of ‘stigma,’ ‘mental health,’ and ‘mental illness’ as Niche Themes should be interpreted with caution. While their centrality score suggests a narrower integration into the wider thematic network, their density indicates conceptual maturity. This positioning may reflect a shift toward the use of more specific descriptors, such as ‘structural stigma,’ ‘self-stigma,’ and ‘perceived discrimination’, which can reduce the frequency of broader keywords without signaling a genuine decline in research attention. In other words, the observed positioning is more likely an artifact of evolving terminology and thematic refinement rather than an indication of diminished scholarly relevance.

In other quadrant, the “*Niche Themes*”, includes *psychiatric* − *disorders, perceived discrimination,* and *mental* − *health*, suggesting these areas are highly developed but may appeal to a more specialized segment of the stigma-focused mental health research community. The quadrant for “*Basic Themes*” contains *mental* − *illness, schizophrenia,* and *attitudes*, reflecting topics with foundational relevance but potentially broader in scope than the depth of research might suggest. Lastly, the “*Motor Themes*” encompass *discrimination, depression,* and *prevalence,* which are central, well − developed areas driving much of the current mental health research narrative.

This thematic map thus provides a strategic overview of the mental health research landscape, indicating the levels of intellectual development and centrality of various research themes, and guides scholars towards understanding the evolution and the current state of the field. In addition, through the co − occurrence clusters, the visualization not only showcases the breadth and depth of stigma-focused mental health research but also illuminates the intricate relationships between various research themes. Each cluster forms a narrative arc, providing insights into different dimensions of mental health, from societal implications to individual experiences, from epidemiological patterns to intersectional identities. This clustering and thematic maps thus offers a roadmap for navigating the complex landscape of stigma-focused mental health literature.

#### Thematic evolution of stigma-focused mental health research narrative

**Fig. 5:**
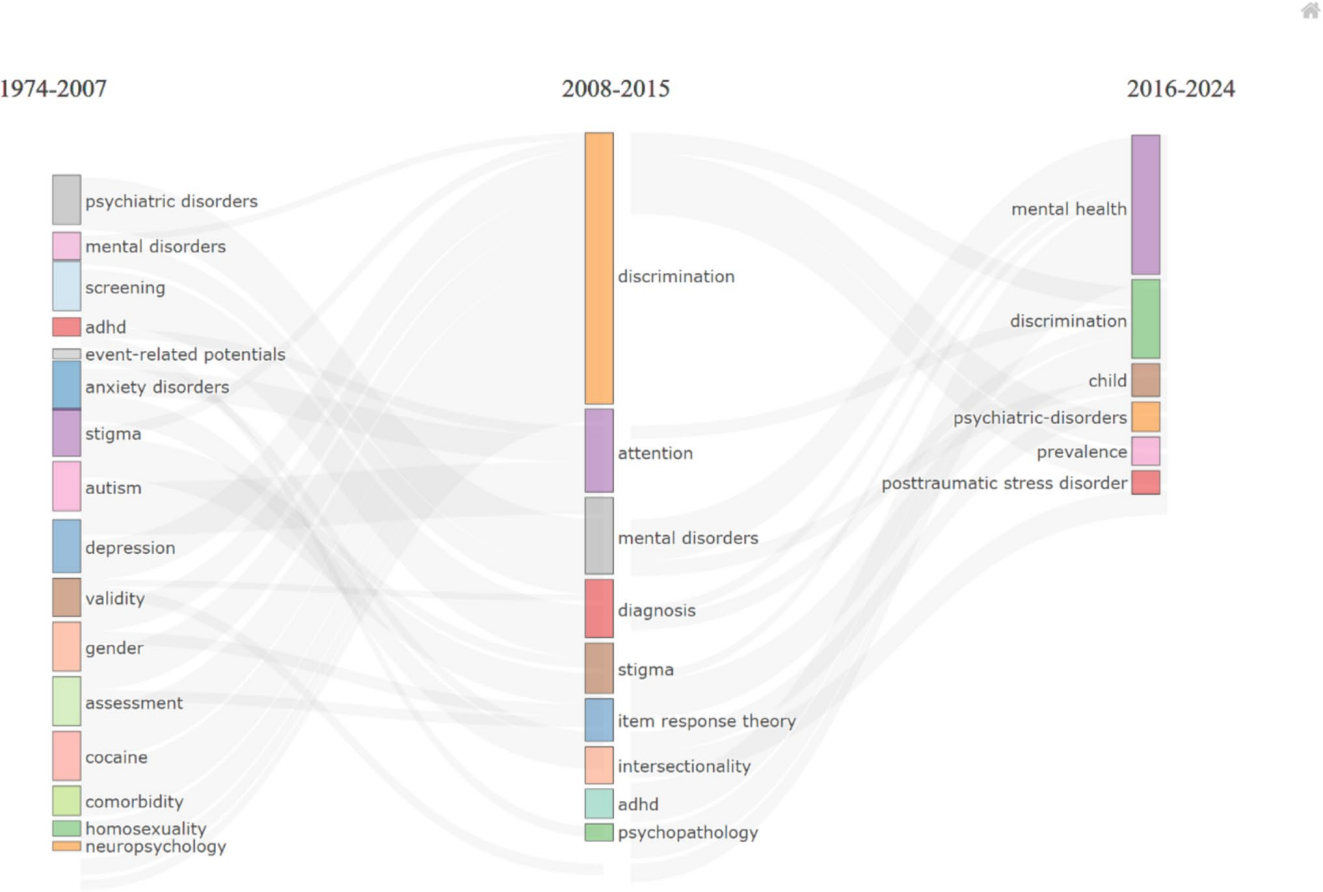
Thematic historiography of stigma-focused mental health research from 1974 to 2024: The diagram illustrates the progression of key research themes over three distinct periods

The bibliometric analysis offers a comprehensive view of the thematic evolution of stigma-focused mental health research over a span of 50 years, delineated into three empirically informed periods based on publication trends, keyword co-occurrence patterns, and citation bursts: the *‘Genesis Period*’ from 1974 − 2007, the *‘Growth Period*’ from 2008 − 2015, and the ‘*Rapid Growth Period’* from 2016 − 2024. These cut-off points were not arbitrarily selected but were informed by both quantitative and conceptual criteria. First, our annual publication growth analysis revealed a marked uptick around 2008, aligning with intensified international stigma-reduction campaigns and the adoption of global mental health frameworks (e.g., WHO’s Mental Health Gap Action Programme [55]). This point marks the end of the Genesis period. Second, the transition to the Rapid Growth phase in 2016 corresponds to several concurrent citation bursts and keyword shifts, particularly the emergence of “child mental health,” “trauma,” and “discrimination” as dominant co-occurring terms. Bibliometrix’s thematic evolution tool and the historiographic analysis of term frequencies (Fig. 5) further confirmed these inflection points. As illustrated in the Fig. 5, we can discern the pivotal themes of each era as well as the thematic interconnections that bridge these periods. The figure’s structure is such that the prominence of a keyword is indicated by the vertical height of its corresponding block, reflecting the term’s frequency of occurrence within that timeframe. The horizontal placement of each block signifies the thematic proximity to other subjects, inferred from the number of co − occurrences, and the grey lines that weave through the blocks represent the strength of thematic ties across the periods, as interpreted by the Bibliometrix tool.

In the *Genesis Period*, we observe foundational themes like mental disorders and stigma taking root, with notable attention to the influence of gender and the onset of conditions like autism and depression. Moving into the *Growth Period*, there is an evident expansion in the research narrative to encompass themes of discrimination and an increased focus on precise assessment and diagnostic techniques. The *Rapid Growth Period* is marked by a surge in research activity, with stigma-focused mental health and discrimination standing at the forefront. Notably, this period witnesses the ascension of child mental disorders and an amplified focus on the prevalence and implications of posttraumatic stress disorder.

These temporal divisions were triangulated by bibliometric performance indicators (e.g., citation bursts identified via Bibliometrix), keyword co-occurrence networks that revealed distinct clusters per period, and thematic shifts captured through the strategic diagram. This evolution not only mirrors the shifts in societal concerns and scientific inquiry but also highlights the growing complexity and interdisciplinary nature of stigma-focused mental health research.

## Discussion

The bibliometric analysis conducted across five decades provides a comprehensive overview of how stigma-focused mental health research has evolved, both thematically and geographically. The identification of three distinct periods—Genesis (1974–2007), Growth (2008–2015), and Rapid Growth (2016–2024)—is supported by shifts in keyword co-occurrence networks, citation bursts, and thematic mapping. The Growth period reflects a diversification of topics, including diagnostic classification (e.g., schizophrenia, bipolar disorder), social consequences (e.g., unemployment, housing instability), and early advocacy for rights-based mental health approaches. This period saw a growing influence from structural stigma theories, particularly in public health frameworks, where stigma is viewed as embedded in societal institutions and policies [25]. The Rapid Growth period (2016–2024) demonstrates a sharp increase in publications, coinciding with heightened awareness of posttraumatic stress disorder, particularly in children and adolescents. The inclusion of child mental health and digital mental health stigma suggests an expanding research scope that incorporates technology-mediated care, trauma-informed frameworks, and youth-centered advocacy. Notably, emerging keywords such as ‘social media,’ ‘cyberbullying,’ and ‘digital health’ indicate a new frontier in stigma research, where online environments are shaping both the perpetuation and mitigation of stigma.

One of the core drivers behind these thematic transformations is the evolving conceptualization of stigma itself. As detailed in Supplementary Table 1, the definition of stigma has expanded considerably, from Erving Goffman’s early interpersonal framework (1963), where stigma was viewed as a socially discrediting attribute, to more contemporary structural models that emphasize systemic, institutional, and policy-based forms of exclusion. The transition from individual-level stigma to structural stigma, as advanced by scholars like Link and Phelan [34] and Hatzenbuehler [25], has significantly shaped research priorities and bibliometric trends.

In the early decades, stigma research primarily focused on visible mental health conditions, societal labeling, and personal experiences of marginalization, particularly surrounding schizophrenia. As stigma theory evolved to incorporate dimensions of power, discrimination, and institutional bias, bibliometric patterns began to reflect this broadening perspective. This is evident in the mid-phase emergence of themes such as self-stigma, intersectionality, and cultural determinants. In the realm of stigma-focused mental health research, the influence of discrimination on stigma-focused mental health spans various forms, as articulated in studies by Vargas et al. [53], who elucidate the compounded effects of multiple discriminations, including racism and heterosexism, which exacerbate mental health issues particularly in LGBTQ racial/ethnic minorities. Complementarily, Vogt Yuan [54] explores the often overlooked aspect of age discrimination and its psychological impacts, highlighting that perceived age discrimination is linked to higher psychological distress, with varying impacts between genders. Both studies stress the importance of addressing multiple and overlapping forms of discrimination in mental health interventions to ensure they are comprehensive and inclusive.

Further complexity is added by Bostwick et al. [5], who examine the interplay of discrimination based on race, gender, and sexual orientation, revealing the nuanced ways in which these intersect to affect mental health among lesbian, gay, and bisexual individuals. This multi − faceted approach to studying discrimination emphasizes a recurring theme also seen in the work of Brown et al. [7], which discusses the specific mental health impacts of racial discrimination over time. Collectively, these studies call for robust, multifaceted public health strategies that recognize and address the specific needs of diverse populations, particularly those facing multiple, intersecting forms of discrimination. The inclusion of various perspectives, including those presented by Thornicroft et al. [48] and the Thornicroft et al. [49], further enriches the discussion by advocating for global and community − level interventions designed to reduce stigma and discrimination in mental health, highlighting the pivotal role of social contact and co − production with people who have lived experiences.

Adding to this discussion, Mossakowski [38] highlights how a robust ethnic identity can buffer the detrimental effects of discrimination on mental health, suggesting a nuanced interplay between cultural belonging and psychological well − being. Similarly, [26] identify significant challenges faced by Asian Americans and Pacific Islanders during the COVID − 19 pandemic, exacerbated by racial discrimination and stigma, necessitating culturally sensitive interventions. These studies higlight the complexity of discrimination’s impact across different societal and ethnic backgrounds and support the need for customized stigma-focused mental health interventions that address these specific contexts. Additionally, the bibliometric analysis by Dikeç et al. [16] provides a broad overview of the shifts in stigma-focused mental health research, notably the increase in studies on discrimination and the stigmatization within psychiatric nursing literature. This analysis emphasizes a growing recognition of the need for stigma reduction strategies and increased cooperation among researchers and institutions to foster advancements in psychiatric care. These findings are echoed in our analysis, which not only confirms the pervasive nature of discrimination but also introduces a new bibliometric framework that traces how stigma-related themes have evolved over five decades. By identifying emerging trends, such as the increasing focus on structural stigma and child mental health, our study offers a new, data-driven perspective on research gaps and future directions in stigma-focused mental health scholarship.

Therefore, our bibliometric study encapsulates half a century of stigma-focused mental health research, charting a thematic shift from early concerns of mental disorders to a pronounced focus on the interplay of discrimination and stigma-focused mental health. The analysis indicates an evolving research landscape, with child mental health and posttraumatic stress disorder gaining momentum, signalling a future where early intervention and personalized mental health care take precedence. The Rapid Growth Period highlights a growing research interest in the interplay between mental health and societal factors, indicating a future where interdisciplinary studies incorporating sociology, psychology, and even economics become more prevalent. The ascent of themes such as child mental health and posttraumatic stress disorder highlights an evolving awareness of the long − term impacts of mental health conditions and the importance of early intervention.

The bibliometric data also revealed an increased focus on child mental health and posttraumatic stress disorder, subjects that have gained considerable academic traction, as noted in the works by Goodman [23], Goodman et al. [22] and Jovanovic et al. [28]. Goodman’s research into child psychopathology (2000) and the Strengths and Difficulties Questionnaire (1999) offer extensive insights into the developmental impacts of psychiatric conditions from a young age. Similarly, Jovanovic (2010) provides critical understanding of the specific biomarkers associated with posttraumatic stress disorder, thereby informing current trends in mental health research that emphasize early diagnosis and intervention. In addition to these, the bibliometric review highlights influential contributions from Schmitt et al. [44] on the psychological well − being effects of perceived discrimination, and Link et al. [35], who explores the enduring effects of stigma in individuals with dual diagnoses. These studies highlight the complex interplay between societal perceptions and individual mental health outcomes.

Furthermore, our analysis highlights the significance of international and interdisciplinary collaborations in enriching stigma-focused mental health research. The global perspective is essential in understanding and addressing the unique and common challenges faced in mental health across different cultural contexts. This globalized approach is evident in the works by Lewis et al. [33] and Moffitt et al. [37], which discuss the global implications of perceived discrimination and the discrepancies in mental health prevalence reported by retrospective and prospective studies. Moreover, the contribution from Corrigan [13] explores the social attributions of stigma-focused mental health research, and Mossman [39] discusses the accuracy of violence predictions in mental health settings. [43] and Jovanovic et al. [28] further explore how early traumatic experiences and fear inhibition mechanisms impact long − term mental health, highlighting the need for a nuanced understanding of these complex phenomena.

Moreover, our global collaboration analysis (Supplementary Fig. 1) reveals a concentration of research output in high-income countries, particularly in North America and Western Europe. This pattern emphasizes a broader issue of geopolitical and sociocultural bias within the field of stigma-focused mental health research. Scholars from low- and middle-income countries (LMICs) remain underrepresented, not necessarily due to a lack of research activity, but often due to systemic barriers such as limited access to funding, infrastructural constraints, language limitations, and the dominance of high-impact journals based in the Global North. This imbalance raises concerns about the generalizability of findings and the potential neglect of context-specific stigma dynamics in LMICs. Addressing this gap requires not only greater inclusion of LMIC-based studies in bibliometric analyses but also the promotion of equitable publishing opportunities and regional or South–South collaborations that amplify diverse perspectives. These steps are essential for building a more inclusive and globally representative knowledge base in mental health research. One actionable pathway to strengthen such South–South collaboration is the establishment of regional open access research platforms dedicated to mental health stigma and discrimination studies. These platforms could facilitate knowledge sharing between LMIC researchers by hosting multilingual repositories of locally generated data, intervention toolkits, and policy briefs. Complementarily, joint funding schemes coordinated by regional development banks or intergovernmental organizations could support collaborative projects between LMIC institutions, ensuring equitable leadership and authorship roles. Additionally, the creation of shared training hubs, offering virtual and in-person capacity-building workshops on bibliometric methods, culturally adapted stigma measurement, and community-based participatory research, would enable sustained skill exchange and foster long-term research networks across the Global South. Such mechanisms would not only address current representation gaps but also embed LMIC-led perspectives into the global discourse on mental health stigma [45, 46].

Thus, understanding how definitions of stigma have evolved over time provides not only a conceptual lens but also a bibliometric rationale for the observed shifts in research focus, co-occurrence clusters, and publication dynamics. Ultimately, the evolution of stigma-focused mental health research, as illuminated by our bibliometric analysis and emphasized by the top ten most cited documents, provides a comprehensive view of the field’s dynamic expansion. Initially focused on fundamental issues related to mental disorders and stigma, the research now embraces a more intricate understanding of discrimination, child mental health, and the interplay with societal transformations. The field is progressively embracing interdisciplinary studies that integrate sociology, psychology, and economics, reflecting a move towards a holistic approach. This expanded perspective is crucial for crafting targeted interventions and policies that effectively address the underlying social determinants of mental health issues, thereby fostering a more equitable and robust approach to stigma-focused mental health care.

## Limitations

This bibliometric study, while extensive, is subject to certain limitations that should be considered when interpreting the findings. Firstly, the analysis was confined to data extracted from specific bibliographic databases, primarily Scopus, Web of Science, PubMed Central, and APA PsycInfo. While these sources provide broad and multidisciplinary coverage, they do not index all relevant publications. Studies available in other databases, institutional repositories, or national archives may not have been captured, which could affect the comprehensiveness of the thematic mapping and the representation of certain regions or disciplines.

Second, the inclusion criteria restricted the dataset to English-language articles. This introduces a language bias, as relevant work published in other languages was excluded. As a result, important contributions, particularly those from non-English-speaking countries, may be underrepresented, potentially overlooking region-specific research patterns and contextually important stigma dynamics. Third, our reliance on peer-reviewed journal publications means that grey literature, books, conference proceedings, and policy reports were excluded. These sources may contain valuable insights, especially regarding emerging themes, local interventions, and practice-based evidence that have not yet entered the formal academic literature. This introduces a publication bias and limits the ability to capture the full spectrum of knowledge on mental health stigma.

Fourth, methodological constraints inherent to bibliometric techniques must be acknowledged. Algorithm-based clustering and keyword co-occurrence analyses, while powerful for detecting patterns, may oversimplify complex and multidimensional scholarly landscapes. Author keyword selection, database indexing practices, and variations in terminology across time and regions can also influence how themes are classified and visualized. Furthermore, temporal trends identified through bibliometric mapping should be interpreted as indicative rather than definitive, as they do not directly measure causal relationships. Lastly, the global collaboration analysis reflects only co-authorship patterns and indexed affiliations. This may underrepresent informal, multidisciplinary, or practice-based collaborations that are not captured in publication metadata, and thus the international research network visualizations may not fully reflect the breadth of collaborative activity in this field.

Despite these limitations, the study has systematically tracked the thematic evolution of stigmatization among stigma-focused mental health research over fifty years, providing a valuable overview of the field’s trajectory. It has identified key thematic shifts and trends that inform current research and practice, setting a foundation for future studies to build upon and ensuring that the discourse around stigma and discrimination in mental health remains robust and progressive. It is imperative, however, that future research incorporates a more diverse range of sources and languages to present a more globally inclusive view and to bridge the gap between research findings and their application in stigma-focused mental health practices.

## Conclusion

This bibliometric study maps out half a century of stigma-focused mental health research, tracing a thematic evolution from initial focus on mental disorders to an intricate examination of discrimination’s role in stigma-focused mental health. It reveals an expanding research landscape where topics like child mental health and posttraumatic stress disorder are gaining prominence, heralding a shift towards early intervention and customized mental health strategies. The emerging trends highlight a deepening interest in the societal influences on mental well − being, calling for future inquiries into the systemic factors that perpetuate stigma and discrimination. The trend towards global collaboration in stigma-focused mental health research emphasizes the possibility of addressing worldwide mental health disparities. Future research directions should also explore emerging avenues such as digital media stigma analytics, which can monitor and address stigma in online environments, and youth-centred intervention designs that leverage technology-mediated engagement to reduce stigma among children and adolescents. This study points towards an interdisciplinary future that integrates digital health technologies and emphasizes mental health equity. Thus, it serves as a guideline for future research, stressing the importance of addressing the multifaceted nature of mental health challenges and pushing forward initiatives aimed at reducing stigma and fostering inclusivity in global mental health discourse and practice.

## Supplementary Information

Below is the link to the electronic supplementary material.

Supplementary file1 (DOCX 1207 KB)

Supplementary file2 (DOCX 18 KB)

Supplementary file3 (DOCX 25 KB)

## Data Availability

No datasets were generated or analysed during the current study.
