# Supplementary material for: Mapping the evolution of stigmatization in mental disorders: A bibliometric analysis from 1974 to 2024
Source: Soc Psychiatry Psychiatr Epidemiol. 2026 Feb 24;61(5):747–63. doi: 10.1007/s00127-025-03003-1 (PMC13156215; doi:10.1007/s00127-025-03003-1)
Supplement: Supplementary file 1 — Supplementary file1 (DOCX 1207 KB) [file 127_2025_3003_MOESM1_ESM.docx]

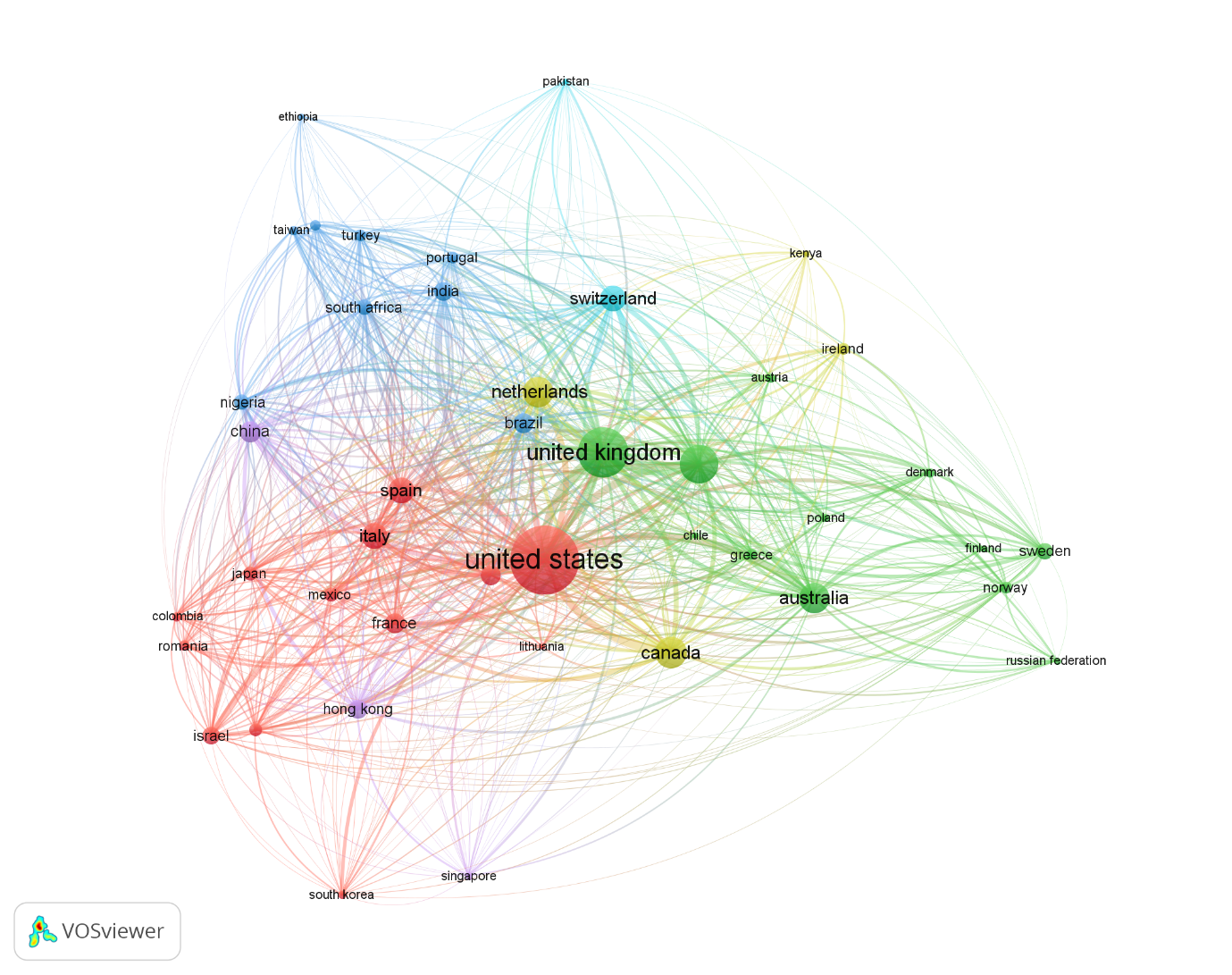


**Supplementary Figure 1.** Network visualization of global research collaborations: This diagram illustrates bibliometric linkages between countries, visualized using VOSviewer. Node size reflects each country’s total link strength within the network, while line thickness indicates the number of collaborative linkages. Diverse colour clusters represent regional or thematic research collaborations.
