# Supplementary material for: Mapping the evolution of stigmatization in mental disorders: A bibliometric analysis from 1974 to 2024
Source: Soc Psychiatry Psychiatr Epidemiol. 2026 Feb 24;61(5):747–63. doi: 10.1007/s00127-025-03003-1 (PMC13156215; doi:10.1007/s00127-025-03003-1)
Supplement: Supplementary file 2 — Supplementary file2 (DOCX 18 KB) [file 127_2025_3003_MOESM2_ESM.docx]

**Supplementary Table 1.** Definitional evolution of ‘Stigma’ and influence on research themes (1974–2024).

| **Period** | **Theoretical Framework** | **Key Conceptualization of Stigma** | **Representative Scholars** | **Influence on Bibliometric Trends** |
| --- | --- | --- | --- | --- |
| 1974–2007 *(Genesis Period)* | Classic Sociological Models | Stigma as a discrediting attribute based on deviation from social norms; interpersonal focus | Erving Goffman (1963) | Early focus on mental illness labelling, societal attitudes, and visibility of conditions like schizophrenia |
| 2008–2015 *(Growth Period)* | Social Psychological and Interactionist Models | Stigma as a process involving labelling, stereotyping, status loss, and discrimination, operating through power dynamics | Link & Phelan (2001), Crocker & Quinn (2003) | Expansion into themes like self-stigma, perceived discrimination, and cultural differences in stigma expression |
| 2016–2024 *(Rapid Growth Period)* | Structural Stigma and Systems-Level Approaches | Stigma embedded in societal structures—laws, institutions, and policies; systemic exclusion and marginalization | Hatzenbuehler (2016), Thornicroft et al. (2022) | Increased focus on intersectionality, structural inequalities, child mental health, trauma, and policy-level analyses |

**Supplementary Table 2.** Strategies for selecting optimal search strings in the systematic review of literature on discrimination and stigmatization in mental disorders.

| **Strategy Step** | **Search String Example** | **Purpose** |
| --- | --- | --- |
| **Strategy 1:** Broad Coverage | (“discrimination” OR “stigmatization”) AND “mental disorders” | To capture a wide array of studies on discrimination or stigmatization within the context of mental disorders, ensuring no relevant literature is missed. |
| **Strategy 2:** Subject−Specific Focus |  |  |
| **Psychology Focus:** | (“discrimination” OR “stigmatization”) AND “mental disorders” AND SUBJAREA(PSYC) | **Psychology**: Targets psychology literature to examine psychological aspects. |
| **Nursing Focus**: | (“discrimination” OR “stigmatization”) AND “mental disorders” AND SUBJAREA(NURS) | **Nursing**: Focuses on nursing literature to explore clinical/caregiving aspects. |
| **Social Sciences Focus**: | (“discrimination” OR “stigmatization”) AND “mental disorders” AND SUBJAREA(SOCI) | **Social Sciences**: Aims at social sciences to study societal and policy dimensions. |
| **Strategy 3**: Keyword Variations and Synonyms | ((“discrimination” OR “stigmatization” OR “bias” OR “prejudice”) AND (“mental disorders” OR “psychiatric disorders” OR “mental illness”)) AND SUBJAREA(PSYC) | Incorporates synonyms and related terms to capture studies using different language for similar concepts, broadening the search to include a more comprehensive set of relevant literature. |
