# Supplementary material for: Mapping the evolution of stigmatization in mental disorders: A bibliometric analysis from 1974 to 2024
Source: Soc Psychiatry Psychiatr Epidemiol. 2026 Feb 24;61(5):747–63. doi: 10.1007/s00127-025-03003-1 (PMC13156215; doi:10.1007/s00127-025-03003-1)
Supplement: Supplementary file 3 — Supplementary file3 (DOCX 25 KB) [file 127_2025_3003_MOESM3_ESM.docx]

**Strategy 1: Broad Coverage**

**Web of Science (n = 1,236):**

TS=((“discrimination” OR “stigmatization*”) AND “mental disorders”) – Publication date: 1973-01-01 to 2024-02-07

**Scopus (n = 3,538):**

TITLE-ABS-KEY ((“discrimination” OR “stigmatization*” ) AND “mental disorders”) AND PUBYEAR > 1973

**PubMed Central (n = 2,510):**

(“discrimination” OR “stigmatization*”) AND “mental disorders” AND (“1974”[Date - Publication] : “2024”[Date - Publication])

**APA PscyInfo (n = 3,508):**

TX ((“discrimination” OR “stigmatization*”) AND “mental disorders”) AND PY > 1973

**Strategy 2: Targeting Psychology** $\boldsymbol{-}$ **A Focused Subject Approach**

**Web of Science (n = 261):**

TS=((“discrimination” OR “stigmatization*”) AND “mental disorders”) AND WC=(Psychology) – Publication date: 1973-01-01 to 2024-02-07

**Scopus (n = 768):**

TITLE-ABS-KEY ((“discrimination” OR “stigmatization*” ) AND “mental disorders”) AND (LIMIT-TO(SUBJAREA, “PSYC”)) AND PUBYEAR > 1973

**PubMed Central (n = 28):**

(“discrimination” OR “stigmatization*”) AND “mental disorders” AND “psychology”[MeSH Terms] AND (“1974”[Date - Publication] : “2024”[Date - Publication])

**APA PscyInfo (n = 18):**

TX ((“discrimination” OR “stigmatization*”) AND “mental disorders”) AND DE “Psychology” (or in a similar way: “AND Classification Code (2220 OR 2223 OR 2224)”) AND PY > 1973

**Strategy 2: Targeting Nursing** $\boldsymbol{-}$ **A Focused Subject Approach**

**Web of Science (n = 33):**

TS=((“discrimination” OR “stigmatization*”) AND “mental disorders”) AND WC=(Nursing) – Publication date: 1973-01-01 to 2024-02-07

**Scopus (n = 254):**

TITLE-ABS-KEY ((“discrimination” OR “stigmatization*” ) AND “mental disorders”) AND (LIMIT-TO(SUBJAREA, “NURS”)) AND PUBYEAR > 1973

**PubMed Central (n = 28):**

(“discrimination” OR “stigmatization*”) AND “mental disorders” AND “nursing”[MeSH Terms] AND (“1974”[Date - Publication] : “2024”[Date - Publication])

**APA PscyInfo (n = 21):**

TX ((“discrimination” OR “stigmatization*”) AND “mental disorders”) AND (“nursing” IN DE) AND PY > 1973

**Strategy 2: Targeting Social Sciences** $\boldsymbol{-}$ **A Focused Subject Approach**

**Web of Science (n = 43):**

TS=((“discrimination” OR “stigmatization*”) AND “mental disorders”) AND WC=(Social Sciences) – Publication date: 1973-01-01 to 2024-02-07

**Scopus (n = 497):**

TITLE-ABS-KEY ((“discrimination” OR “stigmatization*” ) AND “mental disorders”) AND (LIMIT-TO(SUBJAREA, “SOCI”)) AND PUBYEAR > 1973

**PubMed Central (n = 1,100):**

(“discrimination” OR “stigmatization*”) AND “mental disorders” AND “social sciences”[MeSH Terms] AND (“1974”[Date - Publication] : “2024”[Date - Publication])

**APA PscyInfo (n = 5):**

TX ((“discrimination” OR “stigmatization*”) AND “mental disorders”) AND (“social sciences” IN DE) AND PY > 1973

**Strategy 3: Targeting Psychology** $\boldsymbol{-}$ **Keyword Variations and Synonyms**

**Web of Science (n = 1,810):**

TS=((“discrimination” OR “stigmatization” OR “bias” OR “prejudice”) AND (“mental disorders” OR “psychiatric disorders” OR “mental illness”)) AND WC=(Psychology) – Publication date: 1973-01-01 to 2024-02-07

**Scopus (n = 2,817):**

TITLE-ABS-KEY ((“discrimination” OR “stigmatization” OR “bias” OR “prejudice”) AND (“mental disorders” OR “psychiatric disorders” OR “mental illness”)) AND ( LIMIT-TO ( SUBJAREA , “PSYC” ) ) AND PUBYEAR > 1973

**PubMed Central (n = 104):**

((“discrimination” OR “stigmatization” OR “bias” OR “prejudice”) AND (“mental disorders” OR “psychiatric disorders” OR “mental illness”)) AND “psychology”[MeSH Terms] AND (“1974”[Date - Publication] : “2024”[Date - Publication])

**APA PscyInfo (n = 53):**

TX ((“discrimination” OR “stigmatization” OR “bias” OR “prejudice”) AND (“mental disorders” OR “psychiatric disorders” OR “mental illness”)) AND (“psychology” IN DE) AND PY > 1973
